# Author’s Reply

**DOI:** 10.18502/jovr.v15i1.5970

**Published:** 2020-02-02

**Authors:** Mohammad Reza Talebnejad, M. Hossein Nowroozzadeh, Hamideh Mahdaviazad

**Affiliations:** ^1^Poostchi Ophthalmology Research Center, Shiraz University of Medical Sciences, Shiraz, Iran; ^2^Department of Family Medicine, School of Medicine, Shiraz University of Medical Sciences, Shiraz, Iran

We appreciate suggestions presented by Gharebaghi and Heidary regarding our article titled “The Shiraz Pediatric Eye Study – a Population-Based survey of School – Age Children: Rationale, Design, and Baseline Characteristics”.^[1]^ In that study, we aimed to provide a valid source of data via a standard protocol and effective quality assurance strategies. Our article had just described the design, methods, eye examination techniques, and baseline characteristics of the participants. Although the two mentioned studies^[2,3]^ were conducted on a small fraction of the same population, we did not find any scientific justifications to cite them in this publication. They might be cited in future articles from the Shiraz Pediatric Eye Study, if it was deemed necessary.

Their suggestions regarding the prevalence of visual impairments, associations of the race and age with the ocular structures, and the continuing cohort of biometric data are useful. Considering the specific goals of our study (last paragraph of introduction),^[1]^ we have planned to apply the mentioned suggestions and much more in future publications of the Shiraz Pediatric Eye Study.

##  Conflicts of Interest

There are no conflicts of interest.
